# Development and evaluation of gene expression biomarkers for chemical pollution in common frog (*Rana temporaria*) tadpoles

**DOI:** 10.1007/s11356-018-3260-z

**Published:** 2018-09-24

**Authors:** Gunnar Carlsson, Eva Tydén

**Affiliations:** Department of Biomedical Sciences and Veterinary Public Health, Swedish University of Agricultural Sciences, 7028, SE-750 07 Uppsala, Sweden

**Keywords:** *Rana temporaria*, Biomarker, Pollution, Gene expression, cyp1A, Metallothionein, Vitellogenin

## Abstract

Pollutants have been proposed as one factor in the worldwide declines of amphibian species and populations. Applying gene expression analysis of liver RNA in tadpoles would be a possible approach for biomarker measurements to increase knowledge of ecological health in amphibian populations. The major aim of this study was to explore the relevance of applying gene expression analyses of *cytochrome p450* (*cyp1a*), *metallothionein* (*mt*), and *vitellogenin* (*vtg*) in *Rana temporaria* tadpoles. Therefore, tadpoles were exposed for 1 week to β-naphthoflavone (BNF), cadmium chloride (CdCl_2_), and ethinylestradiol (EE2). Primers were developed for RT-qPCR to analyze gene expression in livers. The result showed that the methods for gene expression analyses of *cyp1a*, *mt*, and *vtg* as well as the reference gene β-actin (bact) were successful not only in *R. temporaria* but also in another amphibian, *Rana arvalis*. The gene expression of *cyp1a* was induced by BNF and the gene expression of *mt* was induced by CdCl_2_ but no significant induction was recorded in *vtg* expression after exposure to EE2. Gene expressions varied throughout the tadpole metamorphosis development, in particular for *vtg*. Overall, the use of gene expression of *cyp1a* and *mt* as biomarkers in wild tadpoles seems promising while the use of *vtg* seems less relevant due to high natural variation and low background expression. The study shows that variations in gene expressions between tadpoles of different genetic origin are important to consider when evaluating the data. The present study has thus increased the background knowledge about gene expression applicability as biomarker for tadpoles.

## Introduction

Pollutants have been proposed as one factor in the worldwide declines of amphibian species and populations (Sparling et al. 2001; Hayes et al. 2010). Pollutants can enter aquatic environments from several sources, such as from pesticide application, which may occur in connection with amphibian reproduction and larval development. Amphibians may be attracted to wetlands such as stormwater ponds, potentially impacted of metals and organic compounds (Simon et al. 2009; Brand and Snodgrass 2010; Pohl et al. 2015) and are thus potentially exposed to a large number of chemicals from different sources during the sensitive development period. Reliable biomarkers that reflects the health and pollutant exposure pressure to amphibians are thus of importance. Biomarkers measured in the tadpole can serve as indicators of pollutant levels and potential health impacts that can be connected to factors in the actual site.

Biomarkers indicating pollution are promising tools to increase knowledge of ecological health and can be applied in effect-based environmental monitoring. There are clear evidence of correlations between different biomarkers and adverse effects such as cytochrome p4501A (CYP1A) activity and dioxin-like toxicity (Whyte et al. 2000) as well as vitellogenin (VTG) levels and effects on sexual development (Örn et al. 2003). However, in many aspects, the use of biomarkers has not yet been fully utilized and integrated in monitoring system (Hook et al. 2014). Further development of tools that can screen multiple responses in parallel and monitor harmful effects of pollutants on ecosystem health are further requested (Baker et al. 2014). Examples of established biomarkers used both in experimental studies and environmental monitoring for identification of exposure to environmental pollutants are CYP1A, VTG, and metallothionein (MT), which indicates exposures to organic contaminants such as polyaromatic hydrocarbons, polychlorinated biphenyls and dioxins, and estrogenic compounds and metals, respectively (Hook et al. 2014). The levels of CYP1A, VTG, and MT have traditionally been determined mainly through enzymatic assays and protein quantification, but also through gene expression, i.e., transcript abundance (Sturve et al. 2005; Lehtonen et al. 2006, Fu et al. 2017). The use of mRNA-based measurement of biomarkers instead of protein measurement provides benefits in efficient utilization of small tissue amount and simplified sampling procedure for field conditions (Quirós et al. 2007). Further, several biomarkers can be measured at individual level within the same isolated RNA-sample (Quirós et al. 2007; Baker et al. 2014).

*Rana temporaria* (common frog) and *Rana arvalis* (moor frog) are two relatively prevalent amphibian species in Sweden and northern Europe. We have the last years been studying and sampling tadpoles of these species for different purposes and found them suitable for the use in environmental monitoring. Tadpoles are usually recorded in relatively large numbers and a minor sampling will likely not have a significant impact on future population size, but can add substantially to the knowledge of the general amphibian health. Data obtained from the two *Rana* species might serve as information also for other more endangered species. Applying gene expression analysis of liver RNA would be a possible approach to get biomarker measurements from individual tadpoles even though liver weights only are a few milligram. The aim of the present study was to establish methods for gene expression analyses of *cyp1a*, *mt*, and *vtg* in livers of *R. temporaria* tadpoles and to investigate the relevance of applying these as biomarkers indicating chemical pollution on wild tadpoles. We hypothesized that exposure to β-naphthoflavone (BNF), cadmium chloride (CdCl_2_), and ethinylestradiol (EE2) would increase the hepatic gene expression of *cyp1a*, *mt*, and *vtg*, respectively, in tadpoles of *R. temporaria*.

## Material and methods

### Egg collection and tadpole growth

*R. temporaria* eggs were collected from wild spawnings at three different sites outside Uppsala, Sweden. The distance between the sites (named RT2, RTX, and RT3) were between 5.3 and 23.8 km ensuring genetic diversity between eggs from different sites. Eggs from each site were likely siblings since they were from single clutches. Eggs were raised in site-separated groups in 10 L aquaria with carbon-filtered tap water in room temperature (20 °C). To ensure that sampled eggs were of the correct species, species determination was performed of two individuals from each egg clutch after hatching, according to Palo and Merilä (2003) with slight modifications. Hatched tadpoles were provided duckweed (*Lemna minor*) and Sera micron powder as feed and 80% water exchange was performed twice a week. When the majority of tadpoles from each site had reached stage 35–36 (Gosner 1960) the exposure started, which means that exposure started at different days for tadpoles from different sites.

### Exposure test

One exposure unit consisted of five tadpoles from the same site in 1 L of exposure solution in glass beakers. Exposure solutions consisted of either controls 0.01% DMSO (0.01% DMSO), BNF (300 μg/L), EE2 (0.020 μg/L), or CdCl_2_ (100 μg/L). In addition, one higher concentration of CdCl_2_ was included at exposure start for site RT2 (1000 μg/L). This group was however interrupted after 1 day due to high toxicity and replaced with a group of 10 μg/L CdCl_2_ also used for tadpoles from the other sites. Data obtained from this exposure group were excluded from the study except for the use as reference sample. Stock solutions for all chemicals were prepared in DMSO resulting in 0.01% DMSO in all groups. In total, 60 tadpoles were exposed, i.e., four treatments, tadpoles from three different sites and five tadpoles in each beaker. Exposure solution were totally renewed three times a week (Monday, Wednesday, and Friday) which kept ammonia levels at 4.4 ± 2.5 mg/L before solution change. Temperature were 19.8 ± 0.2 °C throughout the exposure which lasted 7 days and under this period tadpoles were only fed Sera micron.

### Sampling

Tadpoles were euthanized in 0.5 g/L MS-222 buffered with NaHCO_3_. Each tadpole were blotted dry and weighed (BW) to the nearest milligram and measured using a ruler under a stereo microscope to the nearest 0.5 mm. Body length excluding tail (BL), total length including tail (TL), and hind limb length (HLL) was measured as well as determination of the developmental stage. Livers were dissected and stored in Eppendorf tubes with 0.2 mL RNA*later*® (Ambion, Inc), the first 24 h in + 4 °C, later in − 18 °C, according to the manufacturers recommendations.

### Normal development test

The remaining tadpoles not included in the exposure, continued to grow in the original aquaria. To obtain data reflecting the normal development period throughout the metamorphosis process, one tadpole from each site at each developmental stage from stage 34 up to stage 44, were sampled in the same way as the exposed tadpoles.

### RNA isolation, integrity, and concentration determination

RNA was isolated from individual liver samples using NucleoSpin® RNA kit (Macherey-Nagel) according to standard protocol. The RNA concentration and integrity of the samples were measured with Agilent 2100 Bioanalyzer (Agilent Technologies, USA). The RNA samples was later diluted with RNAse free water to obtain a concentration of 10 ng/μL and stored in − 80 °C. As reference sample, a mixture of RNA from five individual livers from the RT3 site exposed to 0.01 μg/L of CdCl_2_, each contributing with the same amount of RNA, was prepared, aliquoted, and stored in − 80 °C also for future use.

### Primer development for *R. temporaria* and *R. arvalis*

Primer3web (primer3.ut.ee) was used for primer design. The aim was to get product sizes between 100 and 200 bp for optimal qPCR analyses. Primer selection for β-Actin (*bact*; reference gene) and Metallothionein (*mt*) was performed with Multiple Sequence Alignment (Clustal Omega) of published sequences for other amphibian species using the NCBI data base to search for conserved regions, thus likely applicable on *R. temporaria* and *R. arvalis*. Published sequences that were included in the alignments were from the following species (including GenBank numbers): *Pelophylax esculentus* (AY973248.1; HE681912.1), *Nanorana parkeri* (XM_018571725.1; XM_018555397.1), *Bufo gargarizans* (EU661596.1), and *Rana catesbeiana* (GQ222412.1). Primers for Cytochrome p450 1A (*cyp1a*) were designed from a *Xenopus tropicalis* sequence (NM_001097344), and then redesigned based on sequenced data of the resulting PCR-product. Vitellogenin (*vtg*) primers were designed directly from an already existing *R. temporaria* sequence (JX997958.1). Primers were ordered and synthesized by Eurofins Genomics (Table 1).

**Table 1:** Information of primers used for different genes in *Rana temporaria* and *Rana arvalis*. F/R is forward and reverse primers, respectively. Also, melting temperatures (*T*_m_) and estimated product size

Gene name	Primer	F/R	Sequence (5′-3′)	*T*_m_ (°C)	Size (bp)
*β-Actin*	*bact*	F	TCAGGTGTCCAGAAGCCCT	58.8	166
		R	GCAATTCCTGGATACATTGTGG	58.4	
*Cytochrome p450 1A*	*cyp1a*	F	TCTCCGTAGCCAATGTCGTT	57.3	109
		R	TGCTGCTCCAAATTTATAGGTCA	57.1	
*Vitellogenin*	*vtg*	F	AGGAAATGTTCAGGTGCGAC	57.3	167
		R	TGCAGGACTGAGAAGGAAGG	59.4	
*Metallothionein*	*mt*	F	GCTCCTGCGGTGATTCCT	58.2	131
		R	GGTATCACATCCCTTTGCACAT	58.4	

Isolated RNA from livers from one *R. temporaria* and one *R. arvalis* tadpole collected earlier from wild populations and stored at − 18 °C in RNA*later* were used as template for primer development. qRT-PCR (protocol, see section 2.7) was performed using the designed primers including non-template controls (NTCs). Melt curve analyses were performed following each reaction to ensure specific product amplification and check for primer-dimer formation. The resulting PCR-products were sequenced by Macrogen and the identity of each sequence was confirmed by NCBI Basic Local Alignment Search Tool (BLAST) to ensure product specificity. Primer efficiency was confirmed by a fourfold dilution series of RNA for each gene which was run in the real-time PCR, where quantification cycle (Ct) number was plotted against logarithm RNA input. For each gene, an appropriate real-time PCR efficiency (E) was confirmed by calculating the equation E = 10[− 1/slope].

### qRT-PCR

Samples (50 ng template RNA per reaction) were run in duplicates in 96-well reaction plates based on the protocol recommended by the manufacturer (QuantiTect® SYBR®Green one-step RT-PCR kit). Deviations from the standard protocol were that a total reaction volume of 25 μL was used instead of 50 μL and that for *vtg*, primer concentration was reduced to half of the recommended. The process was run on a CFX96™ Real-Time PCR Detection System (Bio Rad). The qPCR protocol consisted of reverse transcription 50 °C (30 min), initial denaturation 95 °C (15 min), and 37 cycles of alternating 94 °C (15 s), 55 °C (30 s), and 72 °C (30 s). The protocol ended with determination of melt curves for each PCR-product. All samples were divided in three separate plates for each primer pair. NTCs and reference samples were included in each plate. Mean of the two duplicates were used for further calculations. Gene expression data of reference gene *bact* were calculated according to 2^-ΔCt^ (Livak and Schmittgen 2001) where the result of reference sample were used as calibrator. Expression of target genes (*cyp1a*, *mt* and *vtg*) were calculated as 2^-ΔΔCt^ (Livak and Schmittgen 2001).

### Statistics

Tadpoles from each site separately were exposed in groups of five in each exposure unit. Despite this, every individual was considered as a replicate further in the data analysis due to practical reasons and that interactions between individuals were not considered likely to affect the result in gene expression. Also, variations in data were suspected to be higher in tadpoles from different sites than in tadpoles from the same site. The different response data in the exposure test were analyzed using general linear models (GLM) with treatment and site as factors including the interaction treatment*site. Homoscedasticity of the data was tested by Levene’s test and if required, data was transformed before the GLM. Dunnett’s post hoc test was used to identify treatment effects significantly different to controls. Tukey’s post hoc test was used to compare differences between sites. Spearman’s nonparametric correlation analysis was used to test relationships between developmental stages and gene expressions. Threshold values for determination of induced expressions were applied for the different genes for the identification of data deviating from normality. These were set according to three times the standard deviation above the control mean. The analyses were made in Minitab® 17.1.0 and the level of significance was set to *p* < 0.05.

## Results

### General growth, exposure test

**Fig. 1:**
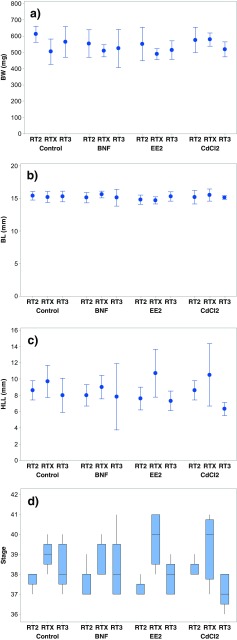
Growth and development parameters in individual *Rana temporaria* tadpoles exposed to BNF, CdCl_2_, EE2, and controls. Tadpoles originate from three different sites (RT2, RTX, and RT3). Graphs show mean ± 95% confidence intervals of body weight (BW, **a**), body length (BL, **b**), hind limb length (HLL, **c**), and a boxplot of developmental stages (**d**)

All tadpoles in the highest concentration of CdCl_2_ (1000 μg/L) which was only started with tadpoles from site RT2 were clearly affected after 1 day of exposure. They were alive, but floating on the surface. Therefore, tadpoles in this group was immediately euthanized and excluded further in the study. One individual exposed to 100 μg/L CdCl_2_ (site RTX) died by accident in connection to water exchange, so this mortality was not regarded as caused by exposure. No other mortality or other apparently toxic effect was recorded among the remaining tadpoles. There were no effects in growth and development responses (BW, BL, TL, HLL, and Stage) in the exposure test between the different treatment groups (*p* ≥ 0.154). However, all these responses except BL (*p* = 0.825) were significantly different between tadpoles from different sites (*p* ≤ 0.018, Fig. 1). Tadpoles from site RT2 had significantly higher BW than tadpoles from RTX (*p* < 0.05) and tadpoles from site RT3 had shorter TL then tadpoles from the other sites (*p* < 0.01). RTX tadpoles were significantly more developed than tadpoles from the other sites, reflected by higher developmental stage (*p* < 0.001) and longer hind limbs (*p* < 0.01). No interaction effects between treatment and site were observed among the growth and development responses (*p* ≥ 0.250).

### Gene expression, exposure test

**Fig. 2:**
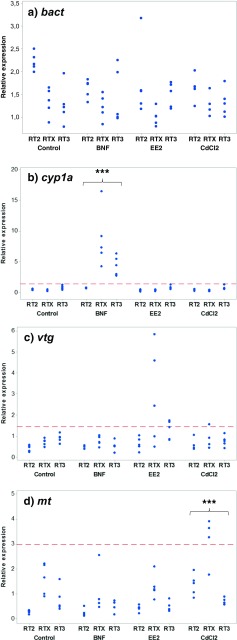
Hepatic gene expressions relative to reference of *bact* (**a**), *cyp1a* (**b**), *vtg* (**c**), and *mt* (**d**) in individual *Rana temporaria* tadpoles exposed to BNF, CdCl_2_, EE2, or controls. Tadpoles originate from three different sites (RT2, RTX, and RT3). *** represents significant differences of exposure treatments as compared to controls (*p* < 0.001; Dunnett’s test). Also, threshold lines are inserted for identification of samples with induced expressions, representing control mean + 3 standard deviations

The relative expression between the different genes in the present study were in order *mt* > *bact* > *cyp1a* > > *vtg*, where *vtg* were close to the detection limit. Some individuals had *vtg* expressions below detection limit and were thus excluded from the vtg data, as lack of products were indicated by observations of the melt curves. The gene expression patterns for *bact*, *cyp1a*, *vtg*, and *mt* in the exposure test are shown in Fig. 2. For all gene expression data, including the reference gene *bact*, there were differences between sites (*p* ≤ 0.002). Tadpoles from site RT2 had significantly higher relative expression of *bact* than tadpoles from the other sites (*p* < 0.01). Tadpoles from all sites differed regarding relative *cyp1a* expression (*p* < 0.001), with highest expression in site RT3 and lowest in RT2. *mt* expression were significantly higher in site RTX (*p* < 0.001) while *vtg* expression were significantly lower in RT2 (*p* < 0.05) compared with the other sites, respectively.

No significantly different expression of *bact* was recorded between treatments (*p* = 0.479) and neither any interaction effect between treatment and site (*p* = 0.263). *cyp1a* expression was affected by treatment (*p* = 0.000). The tadpoles exposed to BNF had a higher expression compared with the controls (*p* < 0.001). Also, there was an interaction effect in *cyp1a* expression between treatment and site (*p* = 0.000). There were no treatment effect recorded on *vtg* expression (*p* = 0.143) and neither any interaction effect between treatment and site (*p* = 0.354). Expression of *mt* was affected by treatment (*p* = 0.000). The tadpoles exposed to CdCl_2_ had a higher expression compared with the controls (*p* < 0.001). Also, there was an interaction effect in *cyp1a* expression between treatment and site (*p* = 0.002).

A positive correlation between stage and *bact* expression was recorded (Spearman’s rho (*ρ*) = 0.367; *p* = 0.046) when including all data in the exposure test. The correlation analyses also revealed some associations between gene expressions expected to be upregulated by chemical exposure and the developmental stage. Correlation analysis between stage and *cyp1a* expression in the tadpoles exposed to BNF also showed a positive correlation (*ρ* = 0.527; *p* = 0.044). Further, *vtg* showed a positive correlation with stage in EE2-exposed tadpoles (*ρ* = 0.659; *p* = 0.008). *mt* expression was however not correlated with stage among CdCl2-exposed tadpoles (*ρ* = 0.520, *p* = 0.057). Threshold values for determination of induced relative expressions were determined to be 1.38, 1.46, and 2.98 for *cyp1a*, *vtg*, and *mt*, respectively (Fig. 2).

### Normal development test

**Fig. 3:**
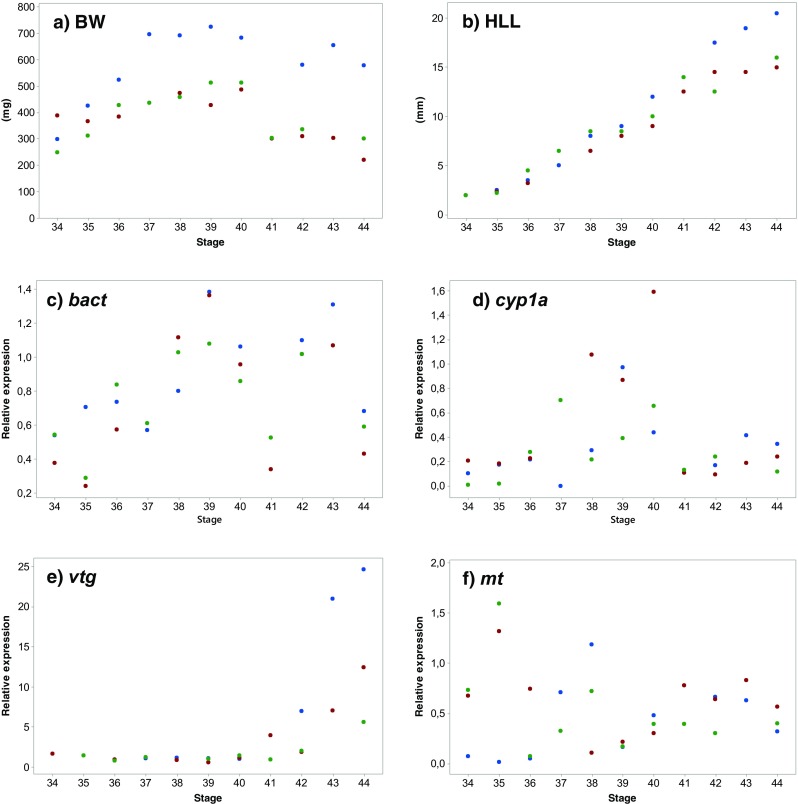
Body weight (BW, **a**), hind limb length (HLL, **b**), and hepatic gene expressions relative to reference of *bact* (**c**) *cyp1a* (**d**), *vtg* (**e**), and *mt* (**f**) in unexposed *Rana temporaria* tadpoles originating from three different sites (RT2, RTX , and RT3), sampled at different stages throughout the metamorphosis

The growth and gene expression data throughout the metamorphosis period between stages 34 and 44 are shown in Fig. 3. Correlation analyses between stage and the different gene expressions revealed positive correlations for *bact* (*ρ* = 0.367; *p* = 0.046) and *vtg* (ρ = 0.675; p = 0,000), but no correlations for *cyp1a* (*ρ* = 0.211; *p* = 0.262) or *mt* (*ρ* = 0.025; *p* = 0.897).

## Discussion

One aim of the present study was to establish methods for gene expression analyses of *cyp1a*, *mt*, and *vtg* in livers of *R. temporaria* tadpoles. This aim was fulfilled since the designed primers including primers for the reference gene *bact* were able to bind to hepatic RNA and amplify expected sequences with the used PCR-protocol, as indicated by runs of the sequenced PCR-products in BLAST. We have also shown that these primers work with the same protocol in another relative species, *R. arvalis*.

Another aim was to investigate the relevance of applying these genes as biomarkers indicating chemical pollution on wild tadpoles. This needs to be considered by several aspects in order to provide a proper assessment. When studying biomarker effects in wild populations, it is fundamental to be aware of how natural variation affects the different measured responses. To include some degree of genetic intraspecific variation, *R. temporaria* eggs were collected from three different sites and were kept separate throughout the whole study. The results from most recorded responses in the exposure test revealed significant variations between tadpoles from different sites. This reflects different growth rates, development progress, and expression of the various biomarkers, which is important to be aware of when applying biomarkers in environmental monitoring programs. Otherwise, there is a risk that a potential difference in gene expression caused by genetic variation is misinterpreted as effects due to chemical exposure. Also, expression of the reference gene (*bact*) showed clear significant differences between sites which also might have influenced the variation between sites in the expressions of other genes. The expression profile of *bact* in unexposed tadpoles throughout the metamorphosis is somewhat inconsistent (Fig. 3c). A positive correlation between stage and *bact* expression was recorded but there are also sudden low expressions at stages 41 and 44, which might imply that *bact* is not a perfect choice as reference gene. Developmental changes in the expression of reference genes during amphibian development have previously been recorded (Hogan et al. 2007; Navarro-Martín et al. 2012). However, *bact* expression was not influenced by the different chemical exposures, which much is considered as the most important condition for a reference gene that is used in connection to biomarker genes for specific chemical impact.

We aimed for exposing the tadpoles to concentrations of chemicals that did not cause any overt toxicity to but high enough to cause inductions in gene expressions. The concentrations of different chemicals were thus not chosen as pollutants in relevant concentrations but rather as model chemicals suspected to impact the gene expressions of interest. A BNF concentration of 300 μg/L was based on a pilot study where we observed a fourfold increase in CYP1A activity (measured using ethoxyresorufin-O-deethylase) on tadpole livers of *R. temporaria* exposed for 1 week to 300 μg/L BNF, but no induction at 30 μg/L (unpublished data). The EE2 concentration was based on literature on data from protein and gene expression analyses on zebrafish and *X. tropicalis* frogs (Örn et al. 2003; Säfholm et al. 2015). We included two starting concentrations of CdCl_2_ (100 and 1000 μg/L) due to varying and limiting toxicity information. The highest concentration was replaced with a lower (10 μg/L) due to toxicity but since there was no toxicity recorded in the 100 μg/L, no analyses were performed in the lower concentration.

As expected, the gene expressions of *cyp1a* were significantly upregulated in tadpoles exposed to BNF in comparison to the controls. However, this upregulation was not as clear in tadpoles from site RT2 as from the other sites (Fig. 2b). The developmental stages for tadpoles from site RT2 were relatively low and tadpoles from site RTX, which had the highest expression, were in slightly higher developmental stages (Fig. 1d). A positive correlation between *cyp1a* expression and stage among the BNF-exposed tadpoles was detected, which might indicate that tadpoles are not fully responding to BNF exposure until later developmental stages. The expressions of *cyp1a* in unexposed tadpoles from stage 34 to 44 showed a non-monotonic pattern with slightly higher expressions between stages 37 and 40 but lower before and after these stages. No clear correlation between stage and *cyp1a* expression was detected among these tadpoles.

We did not reveal any significant *vtg* expression increase in tadpoles exposed to EE2 even though some clearly higher values were obtained in the group from site RTX (Fig. 2c). These tadpoles were the most developed among EE2-exposed tadpoles (stages 40 and 41) and the correlation between stage and *vtg* expression among EE2-exposed tadpoles indicates that tadpoles in later stages are much more responsive to EE2 exposure than in lower stages. Unexposed tadpoles in stages 42 and higher have an even higher expression of *vtg* than the EE2-exposed (Fig. 3e). These findings complicate the development of *vtg* to be used as a biomarker based on the present study. Only the later tadpole stages appears to be sensitive to *vtg* expression and it requires detailed studies to be able to separate between natural and induced variations in *vtg* expression. The *vtg* expressions were also close to the detection limit at lower stages. It has been shown that synthetic estrogens can induce *vtg* expression in newly metamorphosed amphibians (Säfholm et al. 2015). However, studies of *vtg* expression at lower tadpole stages are scarce. The use of VTG as biomarker for estrogenic activity is generally based on males or juveniles, in which the endogenous VTG production is low, but can respond to estrogenic exposure (Wheeler et al. 2005). The increase in *vtg* expression in later developmental stages observed in the present study is probably due to gonad development in females. It has been reported that differentiation of ovaries has its own timing, independent of somatic development in *R. temporaria* and other amphibians (Ogielska and Kotusz 2004). We can thus expect that *vtg* expression do not follow the developmental process and thereby complicates the data interpretation further.

The gene expressions of *mt* were significantly upregulated in tadpoles exposed to CdCl_2_ in comparison to the controls (Fig. 2d). However, much of the data of the tadpoles expected to have induced expressions due to CdCl_2_ exposure are “hidden” within the normal variation of tadpoles from different sites. The expression seems not to be correlated to the developmental stage neither among CdCl_2_ exposed nor in unexposed tadpoles. It is possible that a higher concentration of CdCl_2_ would have revealed a more pronounced induction of *mt*, but the ten-time higher concentration used in the start of the experiment caused clear toxicity which makes it unrealistic for the use as positive control for biomarker development.

The reason for evaluating the method for gene expression analysis in the present study was to apply gene expression data as biomarkers for chemical exposure in wild tadpoles in future studies. As a diagnostic tool, threshold values for determination of induced expressions were applied for the different genes for the identification of data deviating from normality. These thresholds seem to be reasonable in terms of avoiding detecting false positive based on the data in the present study (Fig. 2). However, many individuals that belonged to groups, which were hypothesized to have increased expressions of specific genes, were recorded as negative. Only three data points among CdCl_2_-exposed tadpoles were above the threshold value for *mt* and five data points among EE2-exposed tadpoles were above the threshold value for *vtg*. For tadpoles exposed to BNF, most expressions of *cyp1a* were above the threshold value and thus detected as positive, but also here, all BNF-exposed tadpoles from site RT2 were recorded as negative. These threshold values might need to be further verified by the use in real biomonitoring studies, in particular where chemical analyses can provide data for correlations between effect and exposure. Overall, the use of *cyp1a* as biomarker on *R. temporaria* tadpoles seems to be promising. Wild tadpoles can potentially be exposed to many pollutants causing increases in gene expression of *cyp1a* since other pollutants have been shown to trigger CYP1A activity or gene expression with different potencies than BNF (Smeets et al. 1999). *mt* might be somewhat useful for biomonitoring even though the present study indicates that most individuals exposed to CdCl_2_ were not distinguished from the background. In the present study, *mt* induction was triggered by exposure to CdCl_2_ but there might be other metals or higher metal concentrations that are more potent as *mt* inducers. In carps (*Cyprinus carpio*), mercury and silver exposure was recorded to induce liver mt more as compared with cadmium (Cosson 1993). A similar pattern was observed in 32-h-old zebrafish embryos where mercury were more potent than cadmium, which in turn was more potent than zinc and copper in induction of *mt* gene expression (Chan et al. 2006). There might be influences on *mt* expression also due to metal uptake. All chemicals were exposed via the surrounding water in the present study. It is more likely that organic pollutants but also metals are particle bound or accumulated in plants in wetlands and are thus exposed to tadpoles through feed or by sediment contact which might give other uptake and response patterns. Tadpoles in the present study were exposed for 1 week before gene expression analyses. In natural habitats where tadpoles have been developed and thereby also exposed from their embryo stages, this might also result in different gene expression responses as compared with the present study. The use of *vtg* as biomarker for estrogenic exposure in tadpoles is less promising based on the present study. There are several indications suggesting that the use of *vtg* as biomarker on tadpoles is questionable, due to low gene expression and high natural variation, thereby complicating the detection of affected individuals.

We have shown that the designed primers also work for the use in *R. arvalis*. However, we do not have the background information regarding induction patterns from the positive control substances. To be able to identify deviations from normal expressions also in *R. arvalis*, we might need to perform corresponding exposure studies. Another approach is to hypothesize that both species respond equal to environmental conditions. In samplings from wild populations, tadpoles from both species are often caught from the same ponds and thus correlations in gene expression data between both species can be made to verify or reject this hypothesis.

## Conclusions

We have established methods for gene expression analyses of *cyp1a*, *mt*, and *vtg* in livers of *Rana temporaria* and *Rana arvalis* tadpoles. The hepatic gene expression of *cyp1a* was induced by BNF and the gene expression of *mt* was induced by CdCl_2_ when *R. temporaria* tadpoles were exposed for 1 week to the different chemicals. There was no significant induction in hepatic *vtg* expression in *R. temporaria* tadpoles after exposure to EE2. The use of *cyp1a* and *mt* as biomarkers in wild tadpoles for pollution of organic compounds and metals, respectively, seems promising. *vtg* gene expression data seem less promising as biomarker for estrogenic activity due to high natural variation. The present study shows that variations in gene expressions are high between tadpoles of different genetic origin and that this must be considered in the interpretation of data. By this study, more knowledge is obtained about gene expression applicability as biomarker in tadpoles of *Rana temporaria* for monitoring of pollutant exposure.
